# Exploring Anthracycline-Induced Cardiotoxicity from the Perspective of Protein Quality Control

**DOI:** 10.31083/j.rcm2506213

**Published:** 2024-06-13

**Authors:** Shanshan Li, Weihua Niu, Chunyan Wang, Jie Zhao, Na Zhang, Yue Yin, Mei Jia, Liyan Cui

**Affiliations:** ^1^Department of Laboratory Medicine, Peking University Third Hospital, 100191 Beijing, China; ^2^Core Unit of National Clinical Research Center for Laboratory Medicine, Peking University Third Hospital, 100191 Beijing, China; ^3^Department of Clinical Laboratory, Peking University People’s Hospital, 100041 Beijing, China

**Keywords:** anthracycline-induced cardiotoxicity, protein quality control

## Abstract

Anthracyclines are effective anticancer drugs; however, their use is restricted because of their dose-dependent, time-dependent and irreversible myocardial toxicity. The mechanism of anthracycline cardiotoxicity has been widely studied but remains unclear. Protein quality control is crucial to the stability of the intracellular environment and, ultimately, to the heart because cardiomyocytes are terminally differentiated. Two evolutionarily conserved mechanisms, autophagy, and the ubiquitin-proteasome system, synergistically degrade misfolded proteins and remove defective organelles. Recent studies demonstrated the importance of these mechanisms. Further studies will reveal the detailed metabolic pathway and metabolic control of the protein quality control mechanism integrated into anthracycline-induced cardiotoxicity. This review provides theoretical support for clinicians in the application and management of anthracyclines.

## 1. Introduction

Anthracyclines (ANT) are among the most potent anticancer drugs and are widely used as adjuvants to treat metastatic malignancies, primarily in patients with breast cancer, lymphoma, and pediatric leukemias [1, 2, 3]. However, these drugs exhibit several side effects, particularly cardiotoxicity and myocardial damage. Anthracycline-induced cytotoxicity (AIC) is typically irreversible and persists even after chemotherapy ceases [4, 5]. A wide range of cardiac complications can occur, including congestive heart failure, reduced left ventricular ejection fraction, and irreversible cardiomyopathy [6, 7, 8]. In the clinic, the side-effects of ANT and AIC are controlled by dose restriction while closely monitoring the clinical manifestations of cardiotoxicity. Imaging modalities such as echocardiography are often used to detect cardiotoxicity, which is typically defined as a decrease in the left ventricular ejection fraction ≥10% to a final value of 50% [9]. Which is a unique feature compared with other cardiomyopathies [10]. However, treatments remain limited. Medical therapy is insufficient to reduce these complications, and the condition is associated with high mortality [11, 12]. Therefore, a heart disease subspecialty named cardio-oncology has emerged to handle such cases. Oncologists, cardiologists, and basic scientists in this subspecialty aim to obtain a detailed understanding of the molecular mechanisms of this pathology.

Several mechanisms have been proposed as responsible for AIC, including inhibition of DNA topoisomerase-IIβ [13, 14, 15], oxidative stress [16, 17, 18], and changes in iron metabolism [19]. Topoisomerase inhibition and oxidative stress are the most common mechanisms and involve increases or decreases of certain enzymes or proteins and specific changes in the protein quality control (PQC) system. Thus, balancing the cardiac PQC system has received attention from researchers. The mechanisms underlying the cardiac PQC system and AIC pathogenic proteins require further clarification.

**Fig. 1.**
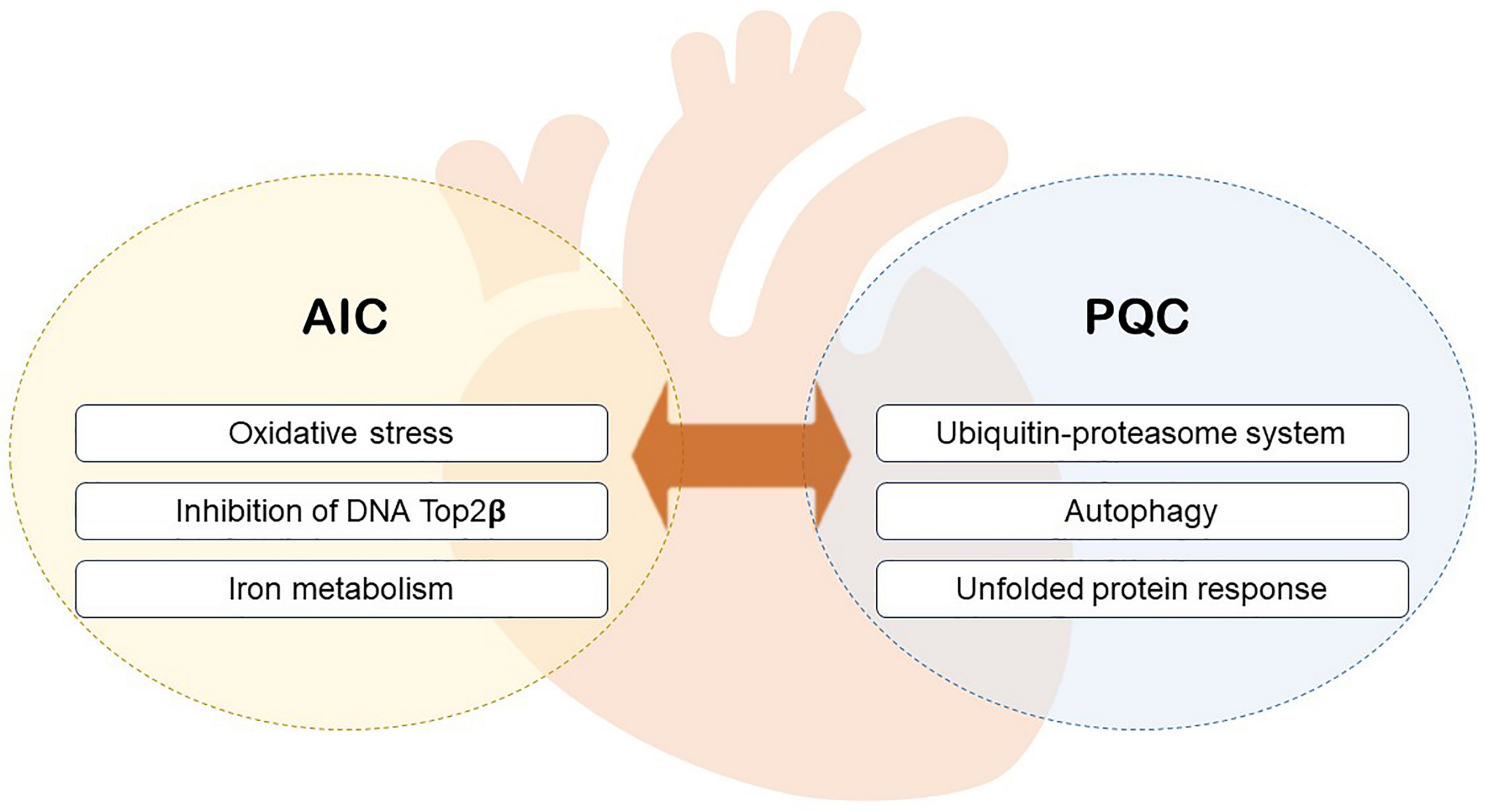
**The relationship between PQC and AIC.** PQC, protein quality control; AIC, anthracycline-induced cytotoxicity.

Intracellular PQC is crucial for maintaining the balance between protein degradation and synthesis [20, 21]. Disappearance of protein homeostasis results in the accretion of misfolded proteins and protein aggregates, ultimately leading to protein toxicity. Previous study clarified the complex adaptive response [22]. Loss or imbalance of the PQC results in numerous diseases, such as neurodegenerative diseases, diabetes, cardiovascular diseases, and cancer. As mitotic cells, myocardial cell possess little ability to replicate in adults [22, 23]. Therefore, maintaining a balanced protein quality minimizes cell dysfunction and death [24, 25]. Additionally, metabolic disorders contribute to cardiovascular disease [26, 27]. PQC is actively involved in regulating cardiac metabolism to maintain these mechanisms. The primary components of PQC are the ubiquitin-proteasome system (UPS), autophagosomal-lysosomal system (autophagy), and unfolded protein response (UPR) [21]. Here, we provide an overview of the PQC of cardiac proteins under AIC while focusing on their interplay with metabolism (Fig. 1).

## 2. Mechanistic Studies of AIC 

**Fig. 2.**
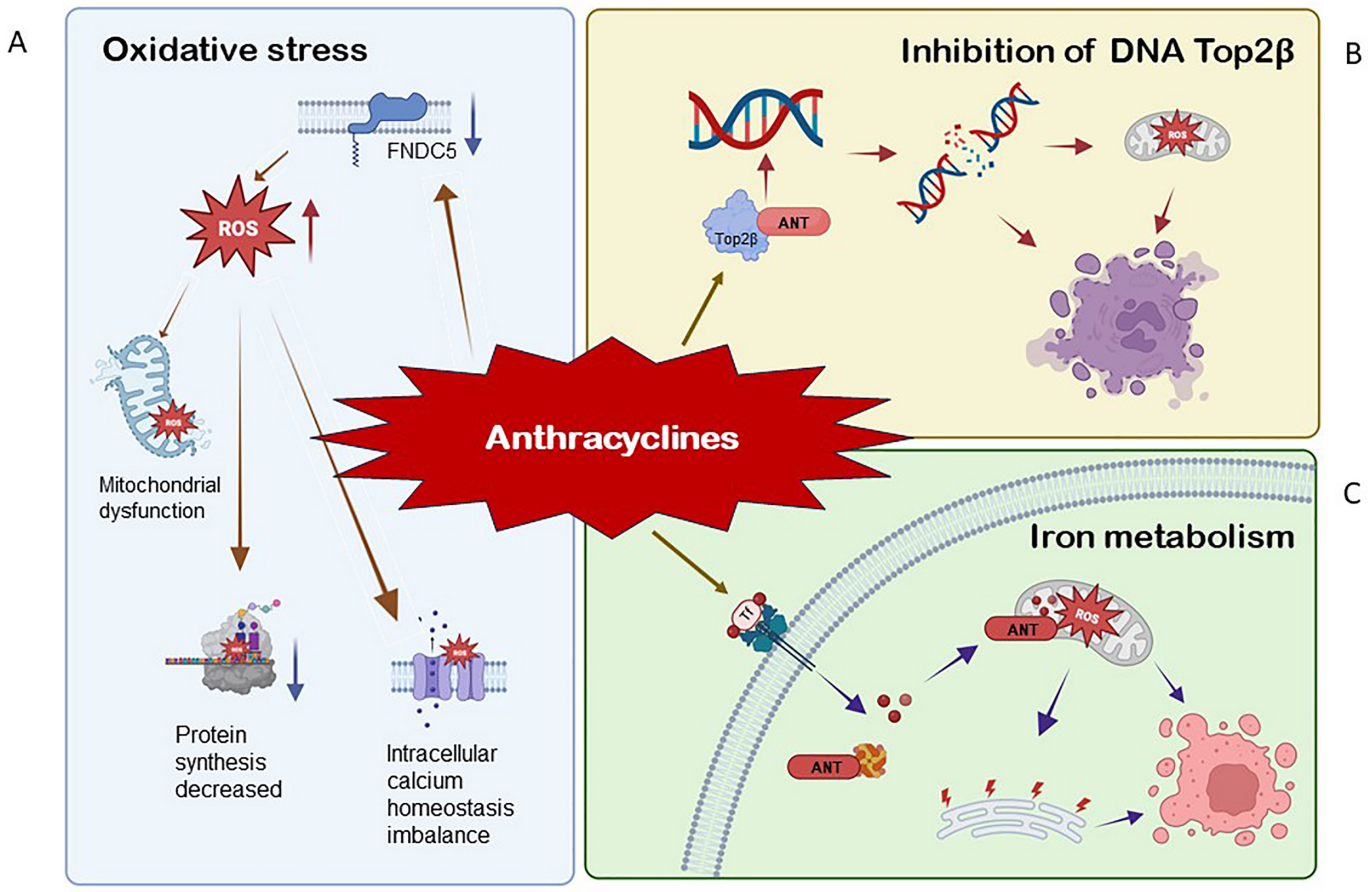
**The mechanism of anthracycline-induced cytotoxicity (AIC).** (A) During the oxidation of NADPH to NADP+, electrons are freed from NADPH to the quinone part of ANT. Semi quinone ANT (SQ-ANT) strongly induced the expression of $O^{2-}$ and $H_{2}$$O_{2}$. ANT can also directly bind to the reductase domain of nitric oxide synthase (NOS), and furthermore, superoxide could react with nitric oxide (NO) to form peroxynitrite (${\mathrm{ONOO}}^{-}$); ANT also prolonged the opening time of endoplasmic reticulum calcium channels, destroyed calcium homeostasis, and then induced apoptosis. (B) ANT directly binds to Top2β in the nucleus, causing sustained DNA damage. (C) In mitochondria, SQ-ANT captures electrons from the cytochrome C and combines with ${\mathrm{Fe}}^{3+}$ to cause the expression of ${\mathrm{OH}}^{-}$, leading to cell death according to apoptosis. NADPH, nicotinamide adenosine dinucleotide phosphate; ANT, anthracyclines; Top2β, topoisomerase-IIβ; ROS, reactive oxygen species.

The exact mechanism(s) via which AIC occurs is unknown. We summarize several of the proposed mechanisms (Fig. 2).

### 2.1 Oxidative Stress

The most commonly proposed mechanism of AIC is the production of reactive oxygen species (ROS), followed by lipid peroxidation [28]. The imbalance between antioxidants and reactive oxygen species (ROS) result in oxidative stress. Low levels of oxidants are indispensable for normal signal transduction; however, high levels of oxidation have been linked to a variety of pathological conditions [29, 30]. When the cumulative dose of doxorubicin (DOX) exceeded 500 mg/$m^{2}$, one of the ANT, it improved oxidative stress [31, 32]. Oxidative stress is caused by the following mechanisms (Table 1, Ref. [33, 34, 35, 36, 37, 38, 39, 40, 41, 42, 43, 44, 45, 46, 47]).

**Table 1.** **Mechanisms inducing oxidate stress**.

Type	Mechanisms	Refs
Reactive oxygen species	The redox cycle at electron transport chain complex I is reduced.	[33, 34]
	High levels of reactive oxygen species may lead to DNA damage, mitochondrial dysfunction, decreased protein synthesis, and intracellular calcium homeostasis imbalance.	[35, 36]
	Oxygen reacts to produce superoxide anion ($O_{2}$^-^), which can be neutralized into superoxide dismutase and combined into stable and low toxic $H_{2}$$O_{2}$.	[37, 38]
	Generate toxic hydroxyl and highly reactive radicals (OH ··).	[39, 40]
Type III fibronectin domain protein 5	A lack of *FNDC5* leads to oxidative damage and increased apoptosis of H9C2 cells under basic conditions.	[41]
Nicotinamide adenosine dinucleotide phosphate (NADPH)	Genetic changes increase NADPH by activating antioxidant transcription factors or through the pentose phosphate pathway.	[42]
	Nox4 is a major cardiac subtype; NADPH oxidases can be activated by a variety of stimuli (such as AngII and periodic stretching of tumour necrosis factor alpha (TNF-α)).	[43, 44]
	Nitric oxide plays an important role in oxidative stress by catalyzing nitric oxide synthase.	[45]
	Nuclear factor erythroid 2-related factor 2 (Nrf2) lack can aggravate cardiac toxicity and cardiac dysfunction.	[46, 47]

#### 2.1.1 Reactive Oxygen Species (ROS) and AIC 

Myocardial cells are seriously rich in mitochondria, which is a major subcellular target of ANT. When compared to other organs, the number of mitochondria in myocardial cells increases by 35%–40% [48, 49]. The redox cycle at the electron transport chain complex I is diminished during ANT therapy, resulting in a substantial amount of ROS and disruption in adenosine triphosphate (ATP) synthesis [33, 34]. In general, high levels of ROS may sensitize cytotoxic signals, leading to DNA damage, mitochondrial dysfunction, decreased protein synthesis, and intracellular calcium homeostasis imbalance [35, 36]. Most ROS are produced in mitochondria. ROS-producing enzymes in mitochondria can convert ANT into semiquinones through a single electron reduction of the quinone group. Semiquinone can easily react with oxygen to produce superoxide anions. Oxygen reacts to produce superoxide anion ($O^{2-}$), which can be neutralized into superoxide dismutase (SOD) and combined to form relatively stable and low-toxicity hydrogen peroxide ($H_{2}$$O_{2}$) [37, 38]. Decomposition enzymes (SOD) are either further transferred to ROS or active nitrogen or further converted to ROS or active nitrogen (RNS) in a series of reactions known as redox cycles [38]. The main concern is that in the iron-catalyzed Fenton reaction, $H_{2}$$O_{2}$ and $O^{2-}$ may also produce toxic hydroxyl and highly reactive radicals (OH ··) [39, 40]. The ROS produced reacts with surrounding mitochondrial biomolecules, including lipids, proteins, and nucleic acids [50, 51].

#### 2.1.2 Type III Fibronectin Domain Protein 5 (FNCD5) Role in Oxidative Stress

The transcription factor peroxisome proliferator-activated receptor (PPAR)- Coactivator 1 (PGC-1) expresses *FNDC5*, a skeletal muscle enrichment protein. The extracellular domain is cleaved to form a circulating 112 amino acid hormone called “Iris” [52, 53]. The expression of fibronectin *FNDC5* was found to be downregulated in ANT-treated mouse heart and myocardial cells. *FNDC5* lack causes oxidative damage and increased apoptosis in H9C2 cells under basic conditions [41]. *In vitro*, it mimics the phenotype of ANT-induced cardiomyopathy. In contrast, *FNDC5* overexpression or irisin treatment reduces ANT-induced oxidative stress and cardiomyocyte apoptosis *in vivo* and *in vitro * [41]. *FNDC5*/Irisin was found to activated protein kinase B (AKT)/mammalian target of rapamycin (mTOR) signaling and reduced ANT-induced cardiomyocyte apoptosis. Additionally, we provided direct evidence that the antioxidant effect of *FNDC5*/Irisin is generated via the protein kinase B/phosphorylated glycogen synthase kinase 3β/phosphorylated Src family tyrosine kinase/nuclear factor erythroid 2-related factor 2 (AKT/GSK3β/FYN/Nrf2) axis in an mTOR-independent manner [41].

#### 2.1.3 Nicotinamide Adenosine Dinucleotide Phosphate (NADPH) and Oxidative Stress

NADPH is another enzyme that helps generate free radicals through redox cycles.

These are a group of plasma membrane related enzymes that serve as a source of ROS [54]. Genetic changes increase NADPH by activating antioxidant transcription factors or via the pentose phosphate pathway (PPP), resulting in high ROS levels, causing cell damage and apoptosis [42]. NADPH oxidase (Noxs), a group of plasma membrane-related enzymes, is one of the most essential ROS sources *in vitro * [55]. Isozymes of Noxs have been identified (Nox1 to 5) [43]. Nox4 is a prominent cardiac subtype, which is expressed in cardiomyocytes, fibroblasts, and endothelial cells; Noxs can be activated by a variety of stimuli (such as AngII, periodic stretching tumour necrosis factor alpha (TNF-α)). It plays a key role in cardiac remodeling [44].

Generally, nitric oxide (NO), as a small molecule substance, also plays a crucial role in oxidative stress by catalyzing nitric oxide synthase (NOS) [45]. Simultaneously, nuclear factor erythroid 2-related factor 2 (Nrf2) also plays an important role in AIC. Nrf2 lack can aggravate cardiac toxicity and cardiac dysfunction. Deoxyribonucleic acid induces oxidative stress, leading to Kelch-like ECH-associated protein-1 (KEAP1)-Nrf2 complex dissociation and an increase in free Nrf2. Nrf2 then enters the nucleus from the cytoplasm, combines with the antioxidant response element in the gene promoter encoding antioxidant enzymes, and upregulates biphasic detoxification enzymes and the expression of downstream antioxidant proteins, thereby reducing oxidative stress Nrf2 [46, 47].

### 2.2 DNA Encoding Topoisomerase-IIβ (Top2β)

Top2β is active in resting non-proliferating cells, including cardiomyocytes. It is currently considered the key mediator of anthracycline-induced cardiotoxicity [56]. Top2β in myocardial cells causes double-stranded DNA breaks. This is necessary to activate the p53 mediated apoptotic cell death pathway and interfere with mitochondrial biogenesis [57]. Several studies have shown that p53 phosphorylation is significantly increased by ANT treatment and deleting p53 or inhibiting the p53 pathway can reduce ANT-induced cardiomyocyte apoptosis and preserve left ventricular ejection fraction [58, 59]. Similarly, dexrazoxane forms a tight complex with the ATPase domains of human Top2α and Top2β. Dexrazoxane-bound Top2 prevents ANT from binding to Top2; therefore, blocking the combination of anthracycline drugs with the Top2 DNA complex may be the mechanism through which dexrazoxane prevents AIC [60].

### 2.3 Changes in Iron Metabolism

Iron deprivation is an iron-dependent lipid peroxidation process, and iron is essential in the occurrence and progression of this process. It is generally believed that excessive iron aggravates AIC [61]. ANT decreases H9C2 myocardial cells activity, while ammonium ferric citrate aggravates it in a concentration-dependent manner [62]. *HFE *gene encodes the HFE protein, which combines with TfR1 and promotes Tf-bound iron uptake. In *HFE* mice treated with ANT, the iron concentration in the heart increases significantly. *HFE* gene mutations cause iron overload in myocardial cells, and myocardial cells are susceptible to iron ptosis and aggravation, indicating the important role of iron metabolism in AIC [63, 64]. Iron transport proteins, which primarily involve cellular iron uptake (TfR1) and storage (ferritin), are regarded as significant indicators of cellular iron homeostasis. For TfR1, most studies suggest that ANT can improve its expression [65, 66]. According to one study, TfR1 induced iron ptosis in cardiotoxicity caused by increased anthracycline iron uptake [67]. After incubation with a particular anti-TfR antibody (12 mg/mL), ANT increased 55Fe absorption. The mechanism of TfR1 upregulation could be related to ANT inhibiting miR-7-5p expression by raising methyltransferase-like 14 (METTL14)-mediated expression of KCNQ1OT1m6A, lowering TfR1 degradation in AC16 cells [67].

### 2.4 Acetylation and AIC

Acetylation is a reversible and highly dynamic modification, which can neutralize and reduce the charge of lysine residues as well as modify the protein structure. As a result, it affects DNA binding affinity, enzyme activity, protein stability, and subcellular localization [68, 69]. Acetylation homeostasis is the main epigenetic mechanism underlying cardiac dysfunction [70]. Proteomic studies have shown that acetylation regulates multiple pathways and sites related to cardiotoxicity by targeting histone deacetylases (HDACs) and histone acetyltransferases (HATs) [71, 72, 73]. Acetylation participates in the cell death pathway via autophagy, oxidative stress, iron metabolism, and other pathways [74].

## 3. PQC and AIC

**Fig. 3.**
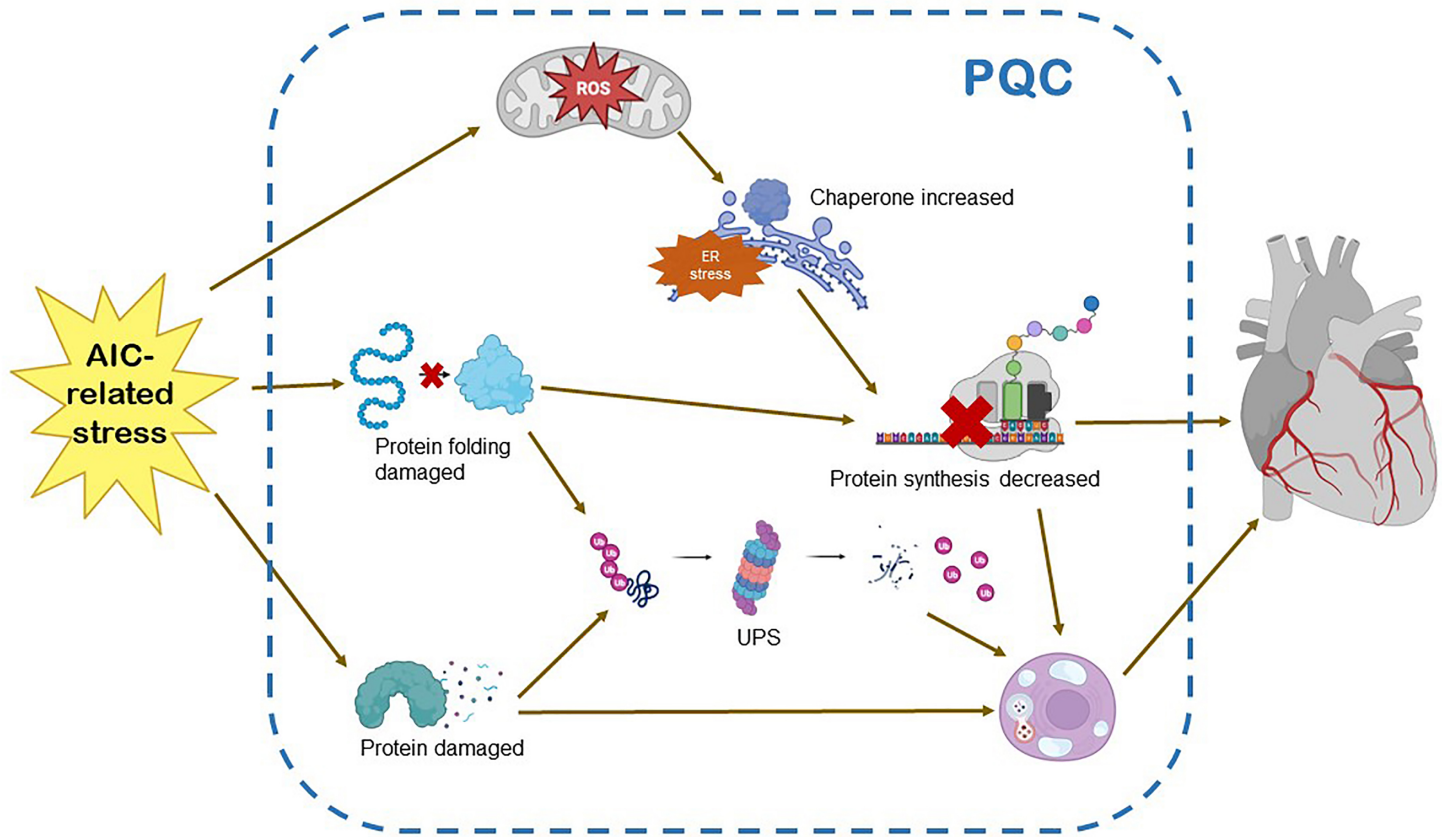
**Protein quality control and AIC.** AIC-related stress on the myocardium triggers increased requirement for protein damage and protein folding. These events, in contrast, sensitize the UPR, the UPS and autophagy. The activation of UPS enhances the transcription and translation of proteins, and causes excessive activation of molecular chaperones. The balance between autophagy and apoptosis is broken. The above will eventually lead to the disorder of protein quality control system and damage of myocardial tissue. UPR, unfolded protein response; UPS, ubiquitin-proteasome system; ER, endoplasmic reticulum; ROS, reactive oxygen species; PQC, protein quality control.

PQC maintains protein homeostasis *in vivo*. Protein homeostasis encompasses gene transcription, mRNA translation, protein post-translational revision, higher-order complex assembly, and protein elimination, which are involved in various aspects of cardiac physiology and pathology. Although considerable progress has been made in our understanding of the regulation of cardiac gene expression, progress in explaining protein quality control is relatively limited [22]. A multitude of cellular mechanisms are used as monitoring systems to ensure protein myocardial cells homeostasis [75, 76]. Autophagy and the UPS are the most essential protein hydrolysis mechanisms for eliminating the cascade reaction of defective organelles and misfolded proteins. These mechanisms involve lysosomes and proteasomes. UPR is an adaptive process that adjusts to endoplasmic reticulum protein-folding stress [77, 78, 79] (Fig. 3).

### 3.1 Autophagy and AIC

Autophagy which is a self-eating process is an evolutionarily conservative process in which cells engulf a small portion of their cytoplasm [80, 81]. Autophagy is an adjusted and highly dynamic procedure, driven by more than 30 autophagy-related proteins (ATGs) [82, 83]. The three forms of autophagy involve transporting substances to lysosomes for degradation. The degree of mega autophagy (hereinafter referred to as autophagy) is the highest among them. This is the best functional research for almost every cell type (Table 2, Ref. [82, 84, 85, 86, 87, 88, 89, 90, 91, 92, 93]).

**Table 2.** **Molecular mechanisms of autophagy resulting in AIC**.

Types	Mechanisms	Refs
Autophagy prom autophagic flow worsens AIC	Improving the expression of Beclin1 protein and mRNA	[82]
	Enhanced expression of different autophagy related markers, such as ATG5, ATG7, and p38	[84, 85]
	Calcium treatment increased ATG7 transcription level	[86]
	Activated AMPK and downregulated p38-MAPK and mammalian target of rapamycin (mTOR) phosphorylation	[87]
	Downregulation of cleaved caspase-3 and LC3-II signaling pathways	[88]
Autophagy inhibits by AIC	Migration inhibitory factor is an indispensable cardioprotective factor against AIC with an underlying mechanism that facilitates autophagolysosome formation	[89]
	Induced accumulation of light chain 3 (LC3)-II and p62	[90]
	Inhibited phosphorylation of AMPK	[91]
	Anthracycline may inhibit vacuolar H+-ATPase activity on lysosomes	[92]
	PI3K γ/AKT activation and PI3K γ inhibition	[93]

AMPK, AMP-dependent protein kinase; p38-MAPK, p38- mitogen-activated protein kinase; PI3K γ, phosphoinositide 3-kinase; ATG, autophagy-related proteins; AIC, anthracycline-induced cytotoxicity; AKT, protein kinase B.

#### 3.1.1 Autophagy Can Worsen AIC

ANT treatment enhances inadaptable cardiac remodeling through *in vivo* and *in vitro* autophagy [94]. According to the study, ANT increases autophagy of cardiomyocytes by promoting the expression of Beclin1 protein and mRNA [82]. AIC enhances the expression of different autophagy-related markers, including ATG5, ATG7, and p38. Other studies have shown that decreased AMP-activated protein kinase (AMPK) and autophagy-initiating kinase unc-51-like kinase 1 (ULK1) expression is involved in AIC pathogenesis [84]. ATG7, the ATP binding and catalytic site of the E1 activating protein, activates two ubiquitin-like proteins, ATG8 and ATG12, which play critical roles in autophagic membrane production during the autophagic membrane formation process [85]. An astounding study demonstrates that ATG7 dependent autophagy is essential for maintaining redox homeostasis and melanocytes biological function, particularly under oxidative pressure [95]. Calcium treatment increased ATG7 transcription level in zebrafish heart and simultaneously activated autophagy in the AIC model [86]. Autophagy inhibition by overexpressing *YAP* down-regulates high mobility group-B1(HMGB1), and silencing ATG7 improves the survival rate of cardiomyocytes treated with ANT [96]. Regarding p38, AIC activated AMPK while downregulating p38-mitogen-activated protein kinase (p38-MAPK) and mTOR phosphorylation resulting in autophagy and initiate the death process of myocardial cells [87]. LC3-II is another key marker of autophagy. Through down-regulating cleaved Caspase-3 and LC3-II signaling pathways, reduced cardiotoxicity was observed in H9C2 cells with ANT stimulation [88]. These studies indicate that autophagy activation is the main reason for myocardial cell programming. Cell death is a reaction to AIC, suggesting that autophagy inhibition is a possible method to prevent AIC.

#### 3.1.2 Autophagy Inhibits AIC

It has been reported that autophagy is inhibited via AIC [97]. Migration inhibitory factor (MIF) serves as an indispensable cardioprotective factor against doxorubicin-induced cardiomyopathy with an underlying mechanism through facilitating auto phagolysosome formation [89]. A rapid high dose of ANT was administered to mice to induce LC3-II and p62 accumulation in the mice hearts [90]. Furthermore, analysis of autophagic flow showed that ANT impaired autophagic lysosomal fusion. Additionally, ANT can inhibit AMPK phosphorylation, which is a positive upstream regulator of autophagy initiation, indicating that ANT inhibits autophagy by reducing its initiation and impairing autophagy flow [91, 92]. Furthermore, ANT inhibits autophagy through PI3K γ/Akt activation, and PI3K γ inhibition reactivates autophagy and protects the heart from AIC [93]. Autophagic flow blockage via ANT may be due to vacuolar H+-ATPase activity inhibition in lysosomes. This in turn causes damaged lysosomes acidification, blocks autophagy lysosome fusion, and produces Beclin1, LC3-II and p62 accumulation [92]. On the contrary, one study showed that ANT treatment enhanced autophagic flux [98]. However, autophagy is firstly stimulated as a compensatory response to cytotoxic stress, it is followed by apoptosis and necrosis at longer exposure times and higher doses. Overall, above studies imply that autophagy is damaged by ANT and that autophagic flow adjustment attenuates myocardial damage.

Whether ANT increases or decreases autophagy, and whether autophagy is protective or harmful to ANT, in still debatable. Different factors may explain these inconsistent results. First, the effect of ANT on autophagy may vary depending on the dose and dose therapy duration. Some AIC disease models have employed an acute high-dose ANT protocol, which could not accurately simulate the clinical situation. Over time, patients exposed to repeated chemotherapy doses develop cardiotoxicity even long after treatment completion. On the contrary, to be more clinically relevant, a chronic low-dose ANT treatment scheme has been established. Its mortality rate is low, which leads to cardiomyopathy development. Second, most studies lack accurate analysis of autophagic flow. LC3-II is a major molecule that directly binds to autophagy or autophagic lysosome cargo and membrane, thus it is considered as a autophagy level marker. LC3-II level may reflect enhanced autophagy formation or autophagy and lysosomes fusion, but not autophagy enhancement. In various studies, the increase in autophagy via ANT does not include autophagy flow analysis, but simply observing autophagy markers LC3-II and p62 expression level may lead to erroneous conclusions.

### 3.2 UPS and AIC

The UPS is the main mechanism for misfolded proteins degradation and intracellular homeostasis promotion [99]. It is a highly regulated multi-step process, starting with ATP-dependent ubiquitin-activating enzymes (E1). Activated ubiquitin is transferred to a conjugated enzyme (E2). Ubiquitin ligase (E3), in turn, combines E2 with the target substrate to catalyze proteins ubiquitination on target lysine and the subsequent extension of polyubiquitin chains. There are approximately two E1 and 40 E2, but over 600 E3 in the human genome, which determine the precise selectivity of protein recognition and degradation [100, 101]. The connected polyubiquitin chain directs the protein to the 26S proteasome, which is a protein complex containing a 20S catalytic core and one or two 19S regulatory cap particles. The ubiquitinated proteins are recognized, deubiquitinated, unfolded from the 19S cap, and transported to the interior of the 20S core for degradation by proteasome peptidases. Finally, the small peptides are released and rapidly hydrolyzed into amino acids. In addition to degrading misfolded proteins, UPS’s target switching to key regulatory molecules is related to cell cycle control, inflammation, and cell death [102]. IκB, p53, and β-catenin are among the real UPS substrates. The UPS plays a significant role in PQC [103, 104].

To evaluate the acute effect of ANT on UPS protein hydrolysis function *in vivo*, green fluorescent protein-dgn (GFP dgn) transgenic mice were treated by DOX intraperitoneal injection (25 mg/kg). At 6 h after doxorubicin injection, GFP dgn protein level but not transcription level decreased significantly. DOX can enhance UPS protein hydrolysis function in intact animals [105]. According to further analysis of its occurrence mechanism, DOX antagonizes UPS endogenous substrates induced by proteasome inhibitors (e.g., β-Accumulation of catenin and c-Jun) in cultured NIH3T3 cells and myocardial cells. DOX activates the UPS by acting directly on both the ubiquitination apparatus and the proteasome [106]. Adriamycin, a key drug in ANT, increases ROS production, which may increase oxidized protein amount. DOX treatment increased UPS substitute protein substrate or endogenous protein degradation, which may be indirectly caused through DOX inadvertently producing ROS on the protein substrate. Oxidized proteins as substrates can indirectly activate UPS proteolysis. Alternatively, as previously discussed, DOX may directly activate the ubiquitination step or the proteasome. [107].

### 3.3 UPR and AIC

Approximately 30% of the proteins in the cytoplasm are processed via the endoplasmic reticulum. These proteins are translated, assembled, and folded in the endoplasmic reticulum (ER) where they are secreted. Additionally, these sequential processes are executed in a coordinated manner. Therefore, cell stressors destroy the homeostasis of the endoplasmic reticulum, resulting in unfolded proteins accumulation, threatening cell survival. To overcome this intracellular crisis known as endoplasmic reticulum stress, cells activate the UPR [108, 109]. Thus, global translation protein synthesis is reduced, UPR gene expression is induced, and redundant proteins are degraded through ER-related degradation and autophagy mechanisms. However, if these adaptive mechanisms cannot function normally due to excessive or continuous stress, apoptosis begins to play a role in protecting host cells [110, 111]. UPR is particularly sensitive to oxidative stress. Mitochondrial ROS production has been revealed to upregulate ER stress markers, resulting in the activation of proadaptive mechanisms that function to decrease protein synthesis, increase chaperone protein production, and downgrade unfolded proteins or sensitize proapoptotic signaling [112, 113]. ANT may promote proteolysis by increasing the UPR through upregulated protein kinase R (PKR)-like ER eIF2 kinase (PERK)-eIF2 signaling and that inhibition of oxidative stress via SS-31 (a protective agent for mitochondrial function) can alleviate ER stress [114]. Moreover, ANT has been proven to induce heart failure due to increased ER stress [115]. Mechanistically, the cardioprotective effects of the UPR have been attributed significantly to consequent enhanced protein folding and the stimulation of ER chaperones.

## 4. Conclusion and Perspectives

AIC is a common disease with various clinical manifestations that compromises the quality of life and overall survival of cancer patients. Treatment strategies are also evolving rapidly. Early detection and preventive treatment have become extremely important. Therefore, it is crucial to identify the pathogenesis of AIC early and then guide clinicians to use drugs. Autophagy, UPR, and UPS are the main mechanisms for the degradation of misfolding. Proteins and defective organelles are used to maintain a functional cell environment. Any dysfunction and disorder in these processes will lead to diseases, such as AIC, myocardial hypertrophy, heart failure, myocardial infarction. In addition, each mechanism is linked to a staggered metabolic cascade. It is necessary to analyze the PQC System and its complex interactions with the metabolic background. We expect that this fascinating biology will drive us to find new therapeutic targets.
